# Supplementary material for: Few Amino Acid Mutations in H6 Influenza A Virus From South American Lineage Increase Viral Replication Efficiency in Poultry
Source: Front Microbiol. 2022 Jul 27;13:953738. doi: 10.3389/fmicb.2022.953738 (PMC9363787; doi:10.3389/fmicb.2022.953738)
Supplement: Supplementary file 1 [file Table_1.docx]

Supplementary Table 1. Replication in trachea and cloaca of wild type and chicken-adapted H6N2 viruses.

| Virus/Group | log_10_ Viral RNA detection^†^ (equivalent EID_50_/ml)^ε^ | | | | | | | | | | |
| --- | --- | --- | --- | --- | --- | --- | --- | --- | --- | --- | --- |
|  | 1 dpi | |  | 3 dpi | |  | 5 dpi | |  | 7 dpi | |
|  | T | C |  | T | C |  | T | C |  | T | C |
| Control | 0/10 (≤ 2) | 0/10 (≤ 2) |  | 0/10 (≤ 2) | 0/10 (≤ 2) |  | 0/4 (≤ 2) | 0/4 (≤ 2) |  | 0/4 (≤ 2) | 0/4 (≤ 2) |
| WT557/H6N2 | 5/10^a^ (2,75) | 1/10^a^ (2,04) |  | 5/10^a^ (2,72) | 1/10^a^ (2,21) |  | 3/4^a^ (3,09) | 0/4^a^ (≤ 2) |  | 0/4^a^ (≤ 2) | 0/4^a^ (≤ 2) |
| 20Ch557/H6N2 | 10/10^b^ (4,96) | 6/10^a^ (2,47) |  | 10/10^b^ (4,52) | 4/10^a^ (2,8) |  | 4/4^a^ (3,31) | 1/4^a^ (2,47) |  | 2/4^a^ (2,06) | 1/4^a^ (2,55) |

T, tracheal swabs; C, cloacal swabs; dpi, days post-infection.

† Number of positive chickens to IAV by RT-qPCR/total number of chickens

ε For statistical purposes, tracheal and cloacal swabs without viral RNA detection (equivalent EID_50_/ml) were given a numeric value of 10^2^ EID_50_/ml, which represents the lowest detectable level of viral RNA with the RT-qPCR used.

Different superscript lowercase letters denote significant differences for number of positive chickens to IAV by RT-qPCR between groups; Chi-Square test, p < 0.05.
